# Enhancing origin prediction: deep learning model for diagnosing premature ventricular contractions with dual-rhythm analysis focused on cardiac rotation

**DOI:** 10.1093/europace/euae240

**Published:** 2024-09-13

**Authors:** Kazutaka Nakasone, Makoto Nishimori, Masakazu Shinohara, Mitsuru Takami, Kimitake Imamura, Taku Nishida, Akira Shimane, Yasushi Oginosawa, Yuki Nakamura, Yasuteru Yamauchi, Ryudo Fujiwara, Hiroyuki Asada, Akihiro Yoshida, Kaoru Takami, Tomomi Akita, Takayuki Nagai, Philipp Sommer, Mustapha El Hamriti, Hiroshi Imada, Luigi Pannone, Andrea Sarkozy, Gian Battista Chierchia, Carlo de Asmundis, Kunihiko Kiuchi, Ken-ichi Hirata, Koji Fukuzawa

**Affiliations:** Division of Cardiovascular Medicine, Department of Internal Medicine, Kobe University Graduate School of Medicine, 7-5-2, Kusunoki-cho, Chuo-ku, Kobe-shi, Hyogo 650-0017, Japan; Division of Cardiovascular Medicine, Department of Internal Medicine, Kobe University Graduate School of Medicine, 7-5-2, Kusunoki-cho, Chuo-ku, Kobe-shi, Hyogo 650-0017, Japan; Division of Molecular Epidemiology, Kobe University Graduate School of Medicine, Hyogo, Japan; Division of Molecular Epidemiology, Kobe University Graduate School of Medicine, Hyogo, Japan; Division of Cardiovascular Medicine, Department of Internal Medicine, Kobe University Graduate School of Medicine, 7-5-2, Kusunoki-cho, Chuo-ku, Kobe-shi, Hyogo 650-0017, Japan; Division of Cardiovascular Medicine, Department of Internal Medicine, Kobe University Graduate School of Medicine, 7-5-2, Kusunoki-cho, Chuo-ku, Kobe-shi, Hyogo 650-0017, Japan; Section of Arrhythmia, Division of Cardiovascular Medicine, Department of Internal Medicine, Kobe University Graduate School of Medicine, Hyogo, Japan; Department of Cardiovascular Medicine, Nara Medical University, Nara, Japan; Division of Cardiovascular Medicine, Hyogo Prefectural Harima-Himeji General Medical Center, Hyogo, Japan; The Second Department of Internal Medicine, University of Occupational and Environmental Health, Kitakyushu, Japan; The Second Department of Internal Medicine, University of Occupational and Environmental Health, Kitakyushu, Japan; Department of Cardiology, Yokohama City Minato Red Cross Hospital, Kanagawa, Japan; Department of Cardiology, Osaka Saiseikai Nakatsu Hospital, Osaka, Japan; Department of Cardiology, Osaka Saiseikai Nakatsu Hospital, Osaka, Japan; Department of Cardiology, Kita-Harima Medical Center, Hyogo, Japan; Department of Cardiology, Kita-Harima Medical Center, Hyogo, Japan; Department of Cardiology, Kita-Harima Medical Center, Hyogo, Japan; Department of Cardiology, Pulmonology, Hypertension, and Nephrology, Ehime University Graduate School of Medicine, Ehime, Japan; Clinic of Electrophysiology, Heart and Diabetes Center NRW, University Hospital of Ruhr-University Bochum, Bochum, Germany; Clinic of Electrophysiology, Heart and Diabetes Center NRW, University Hospital of Ruhr-University Bochum, Bochum, Germany; Department of Cardiology, Ako City Hospital, Hyogo, Japan; Heart Rhythm Management Centre, Postgraduate Program in Cardiac Electrophysiology and Pacing, Universitair Ziekenhuis Brussel—Vrije Universiteit Brussel, European Reference Networks Guard-Heart, Brussels, Belgium; Heart Rhythm Management Centre, Postgraduate Program in Cardiac Electrophysiology and Pacing, Universitair Ziekenhuis Brussel—Vrije Universiteit Brussel, European Reference Networks Guard-Heart, Brussels, Belgium; Heart Rhythm Management Centre, Postgraduate Program in Cardiac Electrophysiology and Pacing, Universitair Ziekenhuis Brussel—Vrije Universiteit Brussel, European Reference Networks Guard-Heart, Brussels, Belgium; Heart Rhythm Management Centre, Postgraduate Program in Cardiac Electrophysiology and Pacing, Universitair Ziekenhuis Brussel—Vrije Universiteit Brussel, European Reference Networks Guard-Heart, Brussels, Belgium; Division of Cardiovascular Medicine, Department of Internal Medicine, Kobe University Graduate School of Medicine, 7-5-2, Kusunoki-cho, Chuo-ku, Kobe-shi, Hyogo 650-0017, Japan; Department of Cardiology, Yodogawa Christian Hospital, 1-7-50, Kunijima, Higashiyodogawa-ku, Osaka-shi, Osaka 533-0024, Japan; Division of Cardiovascular Medicine, Department of Internal Medicine, Kobe University Graduate School of Medicine, 7-5-2, Kusunoki-cho, Chuo-ku, Kobe-shi, Hyogo 650-0017, Japan; Division of Cardiovascular Medicine, Department of Internal Medicine, Kobe University Graduate School of Medicine, 7-5-2, Kusunoki-cho, Chuo-ku, Kobe-shi, Hyogo 650-0017, Japan; Section of Arrhythmia, Division of Cardiovascular Medicine, Department of Internal Medicine, Kobe University Graduate School of Medicine, Hyogo, Japan

**Keywords:** Premature ventricular contraction, Cardiac rotation, Outflow tracts, Artificial intelligence, Algorithm, Deep learning

## Abstract

**Aims:**

Several algorithms can differentiate inferior axis premature ventricular contractions (PVCs) originating from the right side and left side on 12-lead electrocardiograms (ECGs). However, it is unclear whether distinguishing the origin should rely solely on PVC or incorporate sinus rhythm (SR). We compared the dual-rhythm model (incorporating both SR and PVC) to the PVC model (using PVC alone) and quantified the contribution of each ECG lead in predicting the PVC origin for each cardiac rotation.

**Methods and results:**

This multicentre study enrolled 593 patients from 11 centres—493 from Japan and Germany, and 100 from Belgium, which were used as the external validation data set. Using a hybrid approach combining a Resnet50-based convolutional neural network and a transformer model, we developed two variants—the PVC and dual-rhythm models—to predict PVC origin. In the external validation data set, the dual-rhythm model outperformed the PVC model in accuracy (0.84 vs. 0.74, respectively; *P* < 0.01), precision (0.73 vs. 0.55, respectively; *P* < 0.01), specificity (0.87 vs. 0.68, respectively; *P* < 0.01), area under the receiver operating characteristic curve (0.91 vs. 0.86, respectively; *P* = 0.03), and F1-score (0.77 vs. 0.68, respectively; *P* = 0.03). The contributions to PVC origin prediction were 77.3% for PVC and 22.7% for the SR. However, in patients with counterclockwise rotation, SR had a greater contribution in predicting the origin of right-sided PVC.

**Conclusion:**

Our deep learning–based model, incorporating both PVC and SR morphologies, resulted in a higher prediction accuracy for PVC origin, considering SR is particularly important for predicting right-sided origin in patients with counterclockwise rotation.

## Graphical Abstract

**Graphical Abstract:**
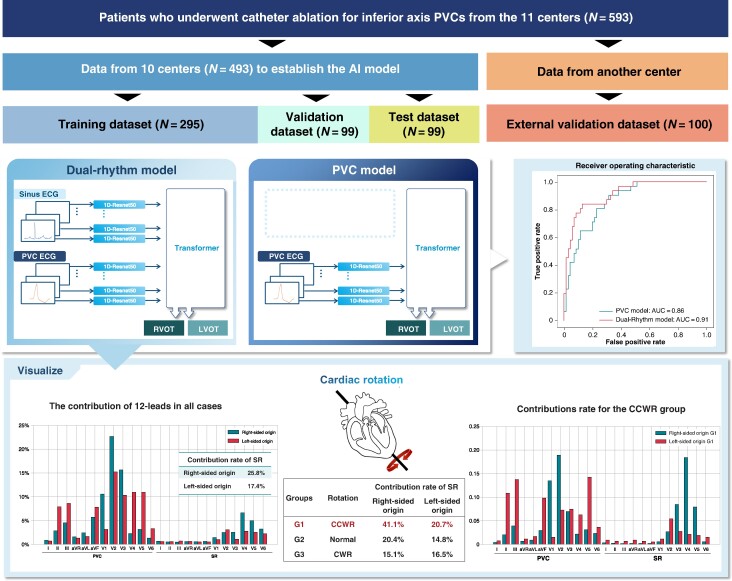

**What’s new?**

- It is unclear how much cardiac rotation affects the 12-lead electrocardiograms (ECGs) of premature ventricular contraction (PVC) from the right or left ventricle.
- This multicentre study, utilizing a hybrid approach combining a Resnet50-based convolutional neural network and a transformer model, aims to predict the PVC origin.
- It compared the dual-rhythm model [incorporating both PVC and sinus rhythm (SR) to the PVC model (using PVC alone)] and quantified the contribution of each ECG lead in predicting the PVC origin for each cardiac rotation.
- The dual-rhythm model demonstrates higher accuracy in origin prediction than the PVC model.
- The contributions to PVC origin prediction were 77.3% for PVC and 22.7% for the SR.
- From the evaluation of the contribution of each ECG lead, considering cardiac rotation is particularly important for predicting right ventricle origin in patients with counterclockwise rotation.

## Introduction

Inferior axis idiopathic premature ventricular contractions (PVCs), originating from various sites including the right or left ventricular outflow tracts (RVOTs/LVOTs), aortic cusp, pulmonary artery, mitral annulus, papillary muscle, and corresponding epicardium, are the most common ventricular arrhythmias.^1^ However, predicting their origin using a 12-lead electrocardiogram (ECG) remains challenging. Catheter ablation is an effective treatment for patients with symptoms or worsened cardiac function owing to frequent PVCs.^2–4^ Identifying the anatomical origin of PVCs before catheter ablation is crucial for determining ablation strategies, reducing procedural time, and preventing complications.^5^

Several algorithms have been proposed to differentiate between the right-sided and left-sided origins of arrhythmias using 12-lead ECGs.^6–18^ However, due to the close and complex anatomy of the outflow tracts and the influence of cardiac rotation, these algorithms have limitations.^19^ In some algorithms, the ECG during sinus rhythm (SR) is utilized to predict the origin by considering cardiac rotation; however, its effectiveness is debatable.^11,12,17^

The global interest in artificial intelligence (AI)'s role in cardiology continues to grow, as highlighted by recent reviews that emphasize its potential in ECG analysis.^20–22^ Several studies have reported the AI-based models that can simultaneously evaluate data from multiple leads to predict PVC origin, offering a more efficient and accurate diagnostic approach.^23–27^ However, these models do not consider cardiac rotation, suggesting potential avenues for improvement.

In this study, we assessed the superiority of the dual-rhythm model (incorporating both SR and PVC) to the PVC model (using PVC alone) and quantified the contribution of individual ECG leads in predicting PVC origin for each cardiac rotational position.

## Methods

### Patient selection and data collection

In this study, we analysed patients who underwent catheter ablation for drug-resistant inferior axis PVCs. A total of 593 patients admitted to 11 centres in Germany, Japan, and Belgium from 2010 to 2022 were enrolled in our study. This included an external validation data set of 100 patients from Belgium. The pre-operative 12-lead ECG data, including SR and PVC, were analysed. Patients with inferior axis PVCs identified by electroanatomic mapping and successfully ablated were included, while patients with implantable cardiac devices or structural heart disease were excluded. Details of data collection, including the number of samples from each facility, are provided in Supplementary material online, *Table S1*. For our analysis, 290 cases of the 12-lead ECG data were exported in digital format from standard ECG machines with a 500 Hz sampling rate and 1.25 μV resolution. The remaining 303 cases were obtained in non-digital formats, such as PDF files, and were manually traced using a pen tablet and Adobe Photoshop (version 24.1, Adobe Inc., San Jose, CA, USA) (see Supplementary material online, *Figure S1*). Both digital and manually traced data were standardized to a sampling rate of 500 Hz, converted to mV, and subsequently transformed into matrix data, ensuring uniformity and consistency across all datasets. The study was approved by the Kobe University Medical Ethical Committee (ethical approval number: B210168).

### Electrocardiogram analysis

Cardiac rotation was assessed based on the transitional zone (TZ) during SR, defined as the position of the precordial leads in which the amplitudes of the R-and S-waves were equal. Normal TZ during SR was defined as the range from leads V3 to V4, and based on TZ position, patients were classified into four groups: Group 1 (G1; counterclockwise rotation, CCWR) if TZ < V3, Group 2 (G2; normal) if V3 ≤ TZ ≤ V4, Group 3 (G3; clockwise rotation, CWR) if V4 < TZ, and Group 4 (G4) if there was right bundle branch block (RBBB) or left bundle branch block. The TZ of the PVC was also analysed; the average TZ score was calculated using a grading system with 0.5-point increments according to the R wave transition site (e.g. TZ < V1 = 0.5, V1 = 1, V1–V2 = 1.5, and V2 = 2 points).^10^ The previously reported algorithm was utilized as a conventional method to calculate the V2 transition ratio based on the R- and S-wave amplitudes in lead V2 and compared the TZ during SR and PVC.^11^

### Mapping and ablation protocol

The catheter ablation was performed without sedation using electroanatomic mapping systems, such as CARTO3 (Biosense Webster, Diamond Bar, CA, USA), Ensite (Abbott, St. Paul, MN, USA), and Rhythmia (Boston Scientific, Marlborough, MA, USA). Intracardiac echocardiography was performed under the direction of the physician. A 6 Fr quadripolar catheter was inserted through the femoral vein and placed through the atrioventricular valve to map the largest His potential. A standard 10-pole diagnostic catheter was positioned in the coronary sinus, and in selected cases, a 2 Fr catheter was inserted into the anterior interventricular vein. Pace and activation mapping were performed with a 7 Fr, 4 mm-tip non-irrigated or a 7.5 Fr, 3.5 mm-tip irrigated ablation catheter in the right and left ventricles and coronary sinus to locate the arrhythmia origin. On observing few PVCs at the beginning of the electrophysiological study, induction was attempted by burst pacing from the RVOT or RV apex with or without isoproterenol infusion and in selected cases with phenylephrine bolus. Pace mapping was performed at the maximum output (1.0 ms, 20 V), and the output was decreased until the pacing could not capture the myocardium. The ablation site was determined by matching pace mapping (>11/12 leads), the earliest bipolar ventricular electrogram preceding QRS onset, with the initial QS morphology for the unipolar ventricular electrogram during PVCs. If a suitable ablation site in the right ventricle was not located, or the ablation failed to abolish the arrhythmia, further mapping of the left ventricle and aortic cusps was performed via a retrograde aortic approach. The radiofrequency current was delivered with an ablation catheter, with a power setting of 25–40 W and 43°C temperature limit. Contact force sensing catheters were used, and the operators targeted a 5–30 g contact force. If the PVCs disappeared or the frequency of arrhythmias diminished after the first 30 s of ablation, energy was delivered continuously for 60 s. Ablation success was defined as the absence of spontaneous or induced PVCs 30 min after the last energy delivery, confirmed by continuous cardiac telemetry over the subsequent 24 h of inpatient care. The origin of PVCs was determined based on the prematurity of QRS onset and response to ablation. When these were not in concordance, the response to ablation was considered the decisive factor. The group in which the prematurity of QRS onset and the response to ablation were concordant was termed the concordance group, while the group in which they were not concordant was termed the non-concordance group. Systemic anticoagulation was achieved with intravenous heparin only whenever left-sided mapping or ablation was performed, targeting a minimum activated clotting time of 350 s.

### Data pre-processing

Data pre-processing involved splitting patient data into three distinct datasets: training data set (60%, 295 patients), validation data set (20%, 99 patients), and test data set (20%, 99 patients). Additionally, an external validation data set consisting of 100 patients was included to further assess the generalizability of the model. For each ECG, we cropped a single heartbeat from the PVC and SR morphologies, centring the crop around the peak of the QRS morphology with a total duration of 0.8 s. Given a sampling rate of 500 Hz, the resulting matrix data for each sample had dimensions of (12, 400). When both the SR and PVC data were input into the model, the two sets of 12-lead ECGs were combined, resulting in a matrix size of (24, 400). Calibration was performed with a baseline set at zero, followed by augmentation including the addition of random noise uniformly distributed up to 0.1 mV, ensuring robustness in our data.

### Model development and training

Our model development was centred around a hybrid approach, combining a 1D Resnet50 convolutional neural network (CNN) model and a transformer model.^28,29^ The Resnet50 model served as an embedding model for each of the 12 leads in the ECG to extract a 256-dimensional feature vector representing the distinct features of each lead. Data from each lead of the 12-lead ECGs were input into a common Resnet50 model sharing the same weights. These features were then fed into a transformer encoder, with the class token output being further processed through a multilayer perceptron to determine the PVC origin. The methodology for this part is identical to that of the vision transformer model.^30^ We developed two variants of this model: the PVC model using only PVC 12-lead ECG data and the dual-rhythm model incorporating both PVC and SR 12-lead ECGs (totalling 24 leads). The transformer model parameters were finely tuned with a batch size of 64 and a learning rate varying log-uniformly from 1e−3 to 1e−7 using the Adam optimizer. Training and validation data sets were employed for parameter tuning to identify the best models. The top five models were selected based on minimal validation losses. These were integrated to construct an ensemble model using soft voting for outcome prediction. Finally, the ensemble model was evaluated using a test data set.

### Decision rationale visualization

Visualizing the decision rationale in our models was a two-phase process. In the first phase, we extracted the attention layers from the transformer-based model that inputs both pre- and post-ECG, identifying which of the 24 leads the model focused on. In the second phase, we employed the GradCAM method in the Resnet50 model to obtain an activation map from the final gradient of the CNN layer.^31^ This map was then combined with the weights of each lead identified in the first phase to produce a comprehensive heat map.

### Statistical analysis

We used precision, recall, specificity, F1-score, area under the curve-receiver operating curve (AUC-ROC), and precision recall-AUC (PR-AUC) as metrics to evaluate the performance of our models. The bootstrap method was applied for statistical testing, calculating 95% confidence intervals (CIs) and *P*-values through two-sided tests to compare the metrics of the different models. The software framework for this study was built in Python 3.8, with PyTorch 1.8 serving as the deep learning (DL) library. The scikit-learn package in Python was used to calculate the various metrics. The trends among groups were analysed using the Jonckheere–Terpstra test.

## Results

### Patient characteristics and procedural data

In this multicentre study, we collected and analysed 12-lead ECG data for SR and PVC from 593 patients across 11 facilities in Japan, Germany, and Belgium. Of these, 493 patients were retrospectively collected from Japan and Germany, serving as an algorithm development data set, while the remaining 100 patients were prospectively collected from Belgium, serving as an external validation data set. Of the cases, 406 (68.5%) were right-sided PVC and 187 (31.5%) were left-sided PVC. Details of the PVC origins are presented in *Table 1*. Out of the entire data set, 303 cases were derived from PDF files, while 290 cases were acquired digitally.

**Table 1:** Distribution of premature ventricular contraction origins in patients

	Sublocations	Algorithm development data set *n* = 493	External validation data set *n* = 100	*P*-value
Right-sided origin	Posterior RVOT	263 (53.3%)	41 (41.0%)	0.032
	Anterior RVOT	56 (11.4%)	25 (25.0%)	<0.001
	Para-Hisian region	18 (3.7%)	3 (3.0%)	0.980
Left-sided origin	LCC	28 (5.7%)	7 (7.0%)	0.781
	RCC	19 (3.9%)	3 (3.0%)	0.903
	RCC-LCC commissure	21 (4.3%)	5 (5.0%)	0.951
	LV summit	51 (10.3%)	6 (6.0%)	0.247
	Aortomitral continuity	10 (2.0%)	4 (4.0%)	0.411
	Anterolateral mitral valve	8 (1.6%)	4 (4.0%)	0.250
	Left anterior fascicle	15 (3.0%)	1 (1.0%)	0.417
	Anterolateral papillary muscle	4 (0.8%)	1 (1.0%)	1.000

The premature ventricular contraction origins were determined based on the prematurity of QRS onset and response to ablation from the endocardium. Values are *n* (%). The *P*-values indicate the significance of differences in sublocation proportions between the data sets, based on a *χ*^2^ test. LCC, left coronary cusp; LV, left ventricle; LVOT, left ventricular outflow tract; RCC, right coronary cusp; RVOT, right ventricular outflow tract.

### Comparison of origin prediction by the dual-rhythm and premature ventricular contraction models

**Figure 1:**
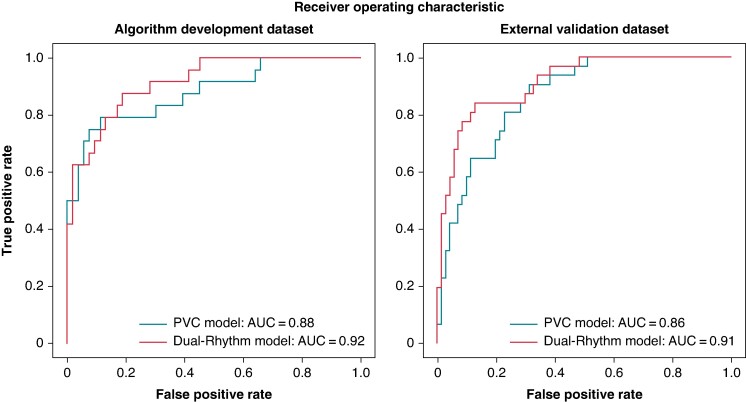
The ROC-AUC of the dual-rhythm and PVC models. In the algorithm development data set, AUC value was 0.92 for the dual-rhythm model and 0.88 for the PVC model. In the external validation data set, AUC value was 0.91 for the dual-rhythm model and 0.86 for the PVC model. AUC, area under the curve; PVC, premature ventricular contraction.

In the algorithm development data set, the dual-rhythm model was superior to the PVC model in accuracy (0.87 vs. 0.78, respectively; *P* < 0.01), precision (0.92 vs. 0.59, respectively; *P* < 0.01), specificity (0.98 vs. 0.76, respectively; *P* < 0.01), AUC-ROC (0.92 vs. 0.88, respectively; *P* = 0.02), and F1-score (0.75 vs. 0.68, respectively; *P* = 0.03) (*Table 2*). In the external validation data set, the dual-rhythm model also outperformed the PVC model, showing higher accuracy (0.84 vs. 0.74, respectively; *P* < 0.01), precision (0.73 vs. 0.55, respectively; *P* < 0.01), specificity (0.87 vs. 0.68, respectively; *P* < 0.01), AUC-ROC (0.91 vs. 0.86, respectively; *P* = 0.03), and F1-score (0.77 vs. 0.68, respectively; *P* = 0.03) (*Table 2*). *Figure 1* illustrates the ROC curves for the algorithm development data set and external validation data set.

**Table 2:** Metrics for the premature ventricular contraction PVC and dual-rhythm models with differences on the algorithm development data set and external validation data set

	Accuracy (95% CI)	Precision (95% CI)	Recall (95% CI)	specificity (95% CI)	AUC_ROC (95% CI)	F1-score^a^ (95% CI)
Algorithm development data set						
PVC model	0.78 (0.74–0.80)	0.59 (0.52–0.67)	0.79 (0.72–0.79)	0.76 (0.71–0.81)	0.88 (0.85–0.92)	0.68 (0.62–0.74)
Dual-rhythm model	0.87 (0.84–0.90)	0.92 (0.91–1.00)	0.63 (0.55–0.72)	0.98 (0.98–1.00)	0.92 (0.89–0.94)	0.75 (0.69–0.81)
Differences	0.10 (0.07–0.15)	0.34 (0.28–0.42)	−0.17 (−0.22 to −0.12)	0.23 (0.18–0.28)	0.04 (0.01–0.06)	0.07 (0.01–0.14)
*P*-value	< 0.01	< 0.01	< 0.01	< 0.01	0.02	0.03
External validation data set						
PVC model	0.74 (0.70–0.79)	0.55 (0.47–0.63)	0.87 (0.81–0.95)	0.68 (0.62–0.75)	0.86 (0.82–0.90)	0.68 (0.60–0.74)
Dual-rhythm model	0.84 (0.81–0.89)	0.73 (0.65–0.83)	0.80 (0.74–0.90)	0.87 (0.82–0.92)	0.91 (0.88–0.95)	0.77 (0.70–0.83)
Differences	0.11 (0.06–0.16)	0.18 (0.11–0.27)	−0.05 (−0.16 to −0.04)	0.19 (0.12–0.26)	0.03 (0.01–0.09)	0.09 (0.02–0.17)
*P*-value	< 0.01	< 0.01	0.03	<0.01	0.03	0.03

AUC-ROC, area under the curve for the receiver operating characteristic; CI, confidence interval; PVC, premature ventricular contraction.

^a^F1 score was calculated as follows: F1-score = 2 × Precision × Recall/(Precision + Recall).

### Comparison of origin prediction by the deep learning model and conventional method

To evaluate the performance of our dual-rhythm model, we compared it with a conventional method that manually measures the R- and S-wave amplitudes. The dual-rhythm model and the conventional method were compared in terms of accuracy (0.87 vs. 0.85, respectively; *P* = 0.88), precision (0.73 vs. 0.83, respectively; *P* = 0.28), recall (0.80 vs. 0.65, respectively; *P* = 0.25), and F1-score (0.77 vs. 0.73, respectively; *P* = 0.67). Notably, the conventional method tended to have higher accuracy for right-sided origin compared to left-sided origin (0.94 vs. 0.65, respectively) (see Supplementary material online, *Table S2*).

### Attention on 12-lead morphology for origin prediction by the deep learning model

**Figure 2:**
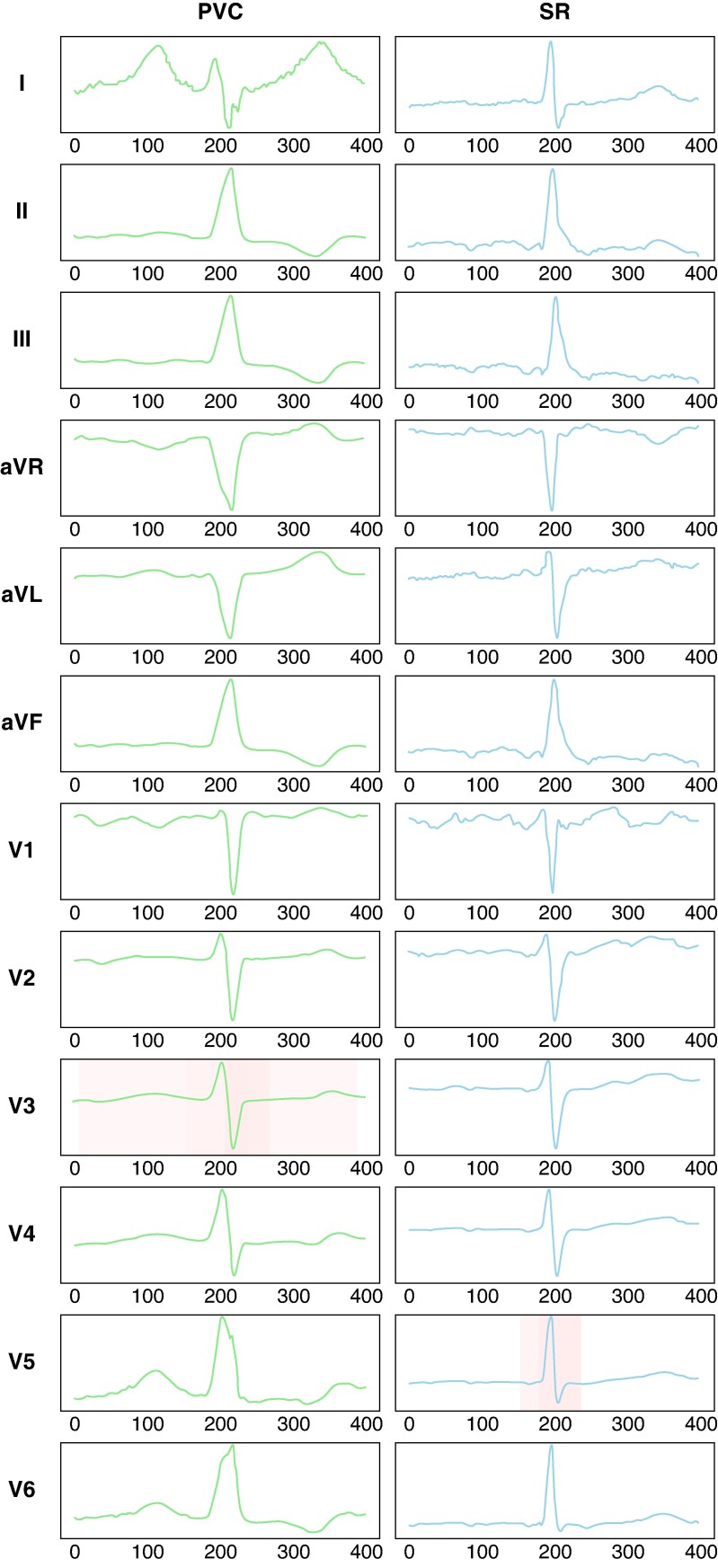
Typical case of attention on 12-lead ECGs of SR and PVC for origin prediction. Visualization of the basis for origin prediction (left, PVC; right, SR). The shaded portions indicate the attention to origin prediction and the percentages. ECG, electrocardiogram; PVC, premature ventricular contraction; SR, sinus rhythm.

**Figure 3:**
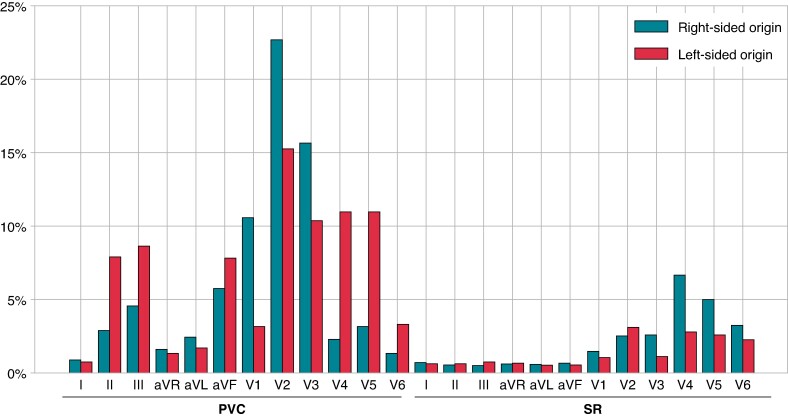
The contribution of each ECG lead to the prediction of the right-sided and left-sided origins in all cases. X-axis represents the ECG leads for PVC and SR, and y-axis represents their percentage contribution. ECG, electrocardiogram; PVC, premature ventricular contraction; SR, sinus rhythm.

*Figure 2* shows a typical case of attention to a 12-lead ECG for origin prediction in the DL model. The PVC was eliminated through radiofrequency application to the RVOT septum. The transition zone of the SR was lead V4 (TZ score = 4). The PVC morphology of lead V3 and SR morphology of lead V5 contributed to the origin prediction. *Figure 3* presents graphs showing the contribution of each ECG lead to the prediction of right-sided and left-sided origins. In all the cases, the contributions to origin prediction were 77.3% for PVC and 22.7% for SR. Specifically, for the right-sided origin, the attention distribution was 74.2% for PVC and 25.8% for SR, whereas for the left-sided origin, it was 82.6% for PVC and 17.4% for SR. Leads V1–3 made significant diagnostic contributions for predicting right-sided origins in PVC, and leads II, III, aVF, and V2–5 for left-sided origins. During SR, the lateral precordial leads, particularly leads V4–6, contributed to the diagnosis.

### Difference in attention for each cardiac rotation

**Figure 4:**
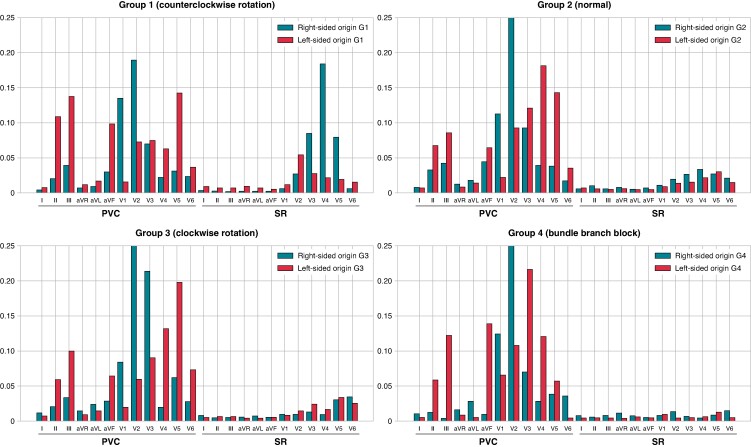
Contribution of each ECG lead to the prediction of the right-sided and left-sided origins for each cardiac rotation. X-axis represents the ECG leads for PVC and SR, and y-axis represents their percentage contribution. Group 1 (G1), patients with TZ < lead V3; Group 2 (G2), V3 ≤ TZ ≤ V4; Group 3 (G3), V4 < TZ; Group 4 (G4), presence of bundle branch block. ECG, electrocardiogram; PVC, premature ventricular contraction; SR, sinus rhythm.

In the classification based on cardiac rotation during SR, the algorithm development data set included 38 (7.7%) patients in G1, 385 (78.1%) in G2, 59 (12.0%) in G3, and 11 (2.2%) in G4. The external validation data set included 11% of patients in G1, 78% in G2, 9% in G3, and 2% in G4. Notably, all G4 patients exhibited an RBBB pattern. The TZ scores for the SR and PVC in each group are presented in *Table 3*. The overall PVC TZ score in the algorithm development data set was 3.7 ± 0.8 for right-sided origin and 1.9 ± 1.1 for left-sided origin. In the external validation data set, the scores were 3.8 ± 0.7 for right-sided origin and 1.8 ± 1.0 for left-sided origin. For both the right-sided and left-sided origins, G1 showed an early TZ reflecting an anterior shift, while G3 showed a late TZ reflecting a posterior shift. The contribution of each ECG leads to origin prediction for each cardiac rotation is shown in *Figure 4*. Particularly, high attention to SR was observed in the right-sided origins in G1. Conversely, in G4, there was negligible focus on SR. In G1, the contribution rate of SR to the prediction of right-sided origins was 41.1%, and that of left-sided origins was 20.7%, marking the highest SR contribution rates to PVC origin predictions among the four groups (see Supplementary material online, *Table S3*).

**Table 3:** Transition zone scores for SR and PVC in each cardiac rotation group based on the origin of right-sided or left-sided PVCs

	Number (%)	Sinus rhythm		PVC	
		Right-sided origin	Left-sided origin	Right-sided origin	Left-sided origin
Algorithm development dataset					
ALL	493	3.4 ± 0.7	3.4 ± 0.8	3.7 ± 0.8	1.9 ± 1.1
G1^a^	38 (7.7%)	1.8 ± 0.2	1.8 ± 0.3	3.0 ± 0.5	1.5 ± 0.7
G2^b^	385 (78.1%)	3.3 ± 0.5	3.4 ± 0.5	3.7 ± 0.7	2.0 ± 1.1
G3^c^	59 (12.0%)	4.6 ± 0.3	4.6 ± 0.2	4.2 ± 0.8	2.2 ± 1.3
G4^d^	11 (2.2%)	0.5 ± 0.0	0.5 ± 0.0	3.8 ± 0.8	1.6 ± 0.8
*P*-value for trend		<0.001	<0.001	<0.001	0.047
External validation data set					
ALL	100	3.4 ± 0.9	3.3 ± 0.9	3.8 ± 0.7	1.8 ± 1.0
G1^a^	11 (11.0%)	1.8 ± 0.4	1.8 ± 0.4	2.8 ± 0.4	1.2 ± 0.4
G2^b^	78 (78.0%)	3.4 ± 0.5	3.5 ± 0.5	3.6 ± 0.5	1.8 ± 1.0
G3^c^	9 (9.0%)	4.7 ± 0.3	4.5 ± 0.0	3.9 ± 0.7	2.0 ± 1.1
G4^d^	2 (2.0%)	1.0 ± 0.0	N.A.	3.0 ± 1.4	N.A.
*P*-value for trend		<0.001	<0.001	<0.001	0.066

The *P*-values for trends among groups G1, G2, and G3 were analysed using the Jonckheere–Terpstra test.

G, group; N.A., not applicable; PVC, premature ventricular contraction; TZ, transition zone.

^a^Patients with TZ < V3.

^b^Patients with V3 ≤ TZ ≤ V4.

^c^Patients with V4 < TZ.

^d^Patients with bundle branch block.

### Comparison of origin prediction based on the concordance and discrepancy between the prematurity of QRS onset and response to ablation

In the external validation data set, 7% of cases showed a discrepancy between the prematurity of QRS onset and the response to ablation. The AUC_ROC for the concordance group was 0.93, while for the non-concordance group, the AUC_ROC was 0.50 (see Supplementary material online, *Figure S2*).

## Discussion

In this study, we utilized a DL model to evaluate whether the accuracy of predicting the origins of inferior axis idiopathic PVCs could be enhanced by incorporating SR. Furthermore, we conducted external validation to affirm the robustness and generalizability of the dual-rhythm model for predicting PVC origins. To the best of our knowledge, this is the first AI-based study to incorporate SR data into the prediction of PVC origin. The main findings of our study are as follows: The dual-rhythm model, which incorporated both the PVC and SR, was significantly superior to the PVC model. The contributions to the prediction were 77.3% from PVC data and 22.7% from SR data. In patients with CCWR, SR had the highest contribution rate to predicting the origin of right-sided PVC.

### Dual-rhythm model for predicting origins from sinus rhythm and premature ventricular contraction data

The RVOT and LVOT are closely positioned and characterized by complex anatomical structures, along with a distinct possibility of being influenced by cardiac rotation.^19^ In this study, we visualized the contribution of the DL model to origin prediction using an attention map and calculated its proportion within a 12-lead ECG. Attention was lower in the limb leads and higher in precordial leads. This indicates a focus on cardiac rotation rather than on the electrical axis during SR. As such, considering the individual-specific cardiac rotation of the SR in our inferences about PVC origins, we believe that our model outperforms traditional models that only use PVC morphologies. In this study, we assessed cardiac rotation using the TZ on a 12-lead ECG. Although imaging modalities such as computed tomography (CT) or magnetic resonance imaging may offer precise evaluations of cardiac rotation, concerns about radiation exposure and the potential side effects of contrast agents make these options less desirable in all cases. A previous study that evaluated the relationship between cardiac rotation on CT and the TZ on ECG found a concordance rate of 80.4%.^32^ This study identified the presence of structural heart diseases, such as dilated cardiomyopathy and hypertrophic cardiomyopathy, as the primary cause of the discrepancies observed. Since our study excluded patients with structural heart disease, we anticipate a higher concordance rate. Therefore, in this study, we utilized ECG-based cardiac rotation, which is more easily obtainable. Betensky *et al.* reported an algorithm combining the morphologies of PVC and SR, using the V2 transition ratio calculated from the proportion of R- and S-waves in lead V2, can accurately differentiate between right- and left-sided PVC origins. In our data set, we also found this method to be effective, although the accuracy slightly decreased for predicting left-sided PVCs.^11^ Our study expands on this approach by evaluating all 12 leads during SR using a DL model. We found that the contribution of the precordial leads was higher than that of the limb leads, further enhancing the accuracy of PVC origin prediction. This suggests that a more comprehensive analysis of precordial leads, and not just lead V2, is crucial for accurately localizing PVC origins.

### Impact of sinus rhythm on the prediction of premature ventricular contraction origins for each cardiac rotation

In this study, we evaluated the changes in the TZ of PVCs for each cardiac rotation and the distribution of attention leads in the dual-rhythm model. In CWR hearts, the TZ for both right-sided and left-sided PVCs moved clockwise, whereas in CCWR hearts, it moved counterclockwise. According to the distribution of attention in the dual-rhythm model, SR had the most significant impact on predicting right-sided PVCs in CCWR cases. For the diagnosis of right-sided PVCs, cardiac rotation towards CWR results in a posterior shift, leading to a later TZ, making the diagnosis easier with only PVC morphology. However, in CCWR hearts, the TZ for right-sided PVCs shifts anteriorly, resulting in an early TZ. This anterior shift can make the PVC morphologies resemble those of left-sided PVCs, potentially leading to a misdiagnosis. In the diagnosis of left-sided PVCs, even in the CWR cases, the PVC remains in an early TZ. This might be because the LVOT is located more centrally in the heart than the RVOT, making it less affected by cardiac rotation.^33^ Our study also found that in patients with RBBB, the SR contributed minimally to origin prediction. This is likely because the RBBB is an issue with the conduction system and does not reflect cardiac rotation.

### The predictive accuracy of premature ventricular contraction origins in the non-concordance group

In this study, the origin of PVCs was determined based on the prematurity of QRS onset and the response to ablation. When these were not in concordance, the response to ablation was considered the decisive factor. In the majority of idiopathic PVCs, ablation is carried out on sites identified using activation and/or pace mapping. However, discrepancies between the results of these methods and the site of successful ablation can complicate the development of an effective ablation strategy. This finding indicates a potential involvement of preferential pathway within the deep myocardium.^34–36^ In this study, preferential pathway was suspected in 7% of cases where there was a mismatch between the prematurity of QRS onset and the response to ablation. When comparing the concordance and non-concordance groups, the non-concordance group tended to have a lower AUC. This observation suggests that the DL model may be predicting the earliest activation site rather than the optimal site for successful ablation.

### Origin prediction model involving transformers

No previous models have incorporated transformers with attention mechanisms to predict PVC origins. In general, origin prediction in DL relies on CNNs, which struggle with long-range relationships. In our study, using transformers with attention mechanisms enabled a better capture of the relationships across the 24 leads of SR and PVC, resulting in an improved prediction model.

Recently, CNN-based models using only sinus rhythm ECGs have been proposed for detecting the presence of PVCs. While these models have shown promise, replacing CNNs with transformers could potentially enhance performance by better capturing long-range dependencies in the ECG data.^37^

### Limitations

In this study, we classified the origins of PVCs into only two classes. The origins of the right-sided or left-sided were determined by the difference in the prematurity of the intracardiac electrograms in electroanatomic mapping and response to ablation. In reality, the origins may be deeper and influenced by preferential pathways. We excluded the patients with implantable cardiac devices or structural heart disease, and our patient cohort did not include those with left bundle branch block. To further enhance the validity and generalizability of our dual-rhythm model, additional prospective external validation is warranted. Furthermore, the model should be refined to classify PVC origins into more than two categories, incorporating more complex anatomical structures and conduction pathways.

## Conclusions

We developed a DL-based model to predict the origins of PVC by incorporating both PVC and SR morphologies. Our findings underscore the importance of considering SR for achieving higher prediction accuracy, especially for predicting the right-sided origin in patients with CCWR.

## Supplementary Material

euae240_Supplementary_Data

## Data Availability

Fully anonymized data will be shared where patient consent allows, on reasonable request to the corresponding author. The code used in this study is available on GitHub at the following URL: https://github.com/macostrail/dual-rhythm-model. This repository contains all scripts, data processing steps, and model implementation details necessary for reproducing the results reported in this paper.
