# Supplementary material for: Enhancing origin prediction: deep learning model for diagnosing premature ventricular contractions with dual-rhythm analysis focused on cardiac rotation
Source: Europace. 2024 Sep 13;26(10):euae240. doi: 10.1093/europace/euae240 (PMC11448329; doi:10.1093/europace/euae240)
Supplement: euae240_Supplementary_Data [file euae240_supplementary_data.docx]

**Supplemental Table 1.** **Data collection details from multiple centers**

| No. | Institution | N |
| --- | --- | --- |
| 1 | Division of Cardiovascular Medicine, Department of Internal Medicine,  Kobe University Graduate School of Medicine, Hyogo, Japan | 99 |
| 2 | Department of Cardiovascular Medicine,  Nara Medical University, Nara, Japan | 95 |
| 3 | Division of Cardiovascular Medicine,  Hyogo Prefectural Harima-Himeji General Medical Center, Hyogo, Japan | 64 |
| 4 | The Second Department of Internal Medicine,  University of Occupational and Environmental Health, Kitakyushu, Japan | 48 |
| 5 | Department of Cardiology,  Yokohama City Minato Red Cross Hospital, Kanagawa, Japan | 47 |
| 6 | Department of Cardiology,  Osaka Saiseikai Nakatsu Hospital, Osaka, Japan | 42 |
| 7 | Department of Cardiology,  Kita-Harima Medical Center, Hyogo, Japan | 33 |
| 8 | Department of Cardiology, Pulmonology, Hypertension, and Nephrology,  Ehime University Graduate School of Medicine, Ehime, Japan | 32 |
| 9 | Clinic of Electrophysiology, Heart and Diabetes Center NRW,  University Hospital of Ruhr-University Bochum, Bochum, Germany | 28 |
| 10 | Department of Cardiology,  Ako City Hospital, Hyogo, Japan | 5 |
| 11 | Heart Rhythm Management Centre, Postgraduate Program in Cardiac Electrophysiology and Pacing, Universitair Ziekenhuis Brussel – Vrije Universiteit Brussel, Brussels, Belgium | 100 |

**Supplemental Table 1. Data collection details from multiple centers**

**Supplemental Table 2.**

|  | Accuracy | | Precision | Recall | F1-Score^‡^ |
| --- | --- | --- | --- | --- | --- |
|  | Right-sided origin | Left-sided origin |  |  |  |
| Dual-Rhythm Model  (External validation dataset) | 0.87 (0.80-0.94) | 0.81 (0.65-0.94) | 0.73 (0.65-0.83) | 0.80 (0.74-0.90) | 0.77 (0.70-0.83) |
| Conventional method | 0.94 (0.88-0.99) | 0.65 (0.48-0.81) | 0.83 (0.67-0.96) | 0.65 (0.46-0.82) | 0.73 (0.57-0.86) |
| p-value | 0.11 | 0.20 | 0.28 | 0.25 | 0.67 |

**Supplemental Table 2. Comparison of Origin Prediction by the DL model and Conventional Method.**

^†^ F1 score was calculated as follows: F1-Score = 2 × Precision × Recall / (Precision + Recall)

**Supplemental Table 3.**

|  | Algorithm development dataset | | | | External validation dataset | | | |
| --- | --- | --- | --- | --- | --- | --- | --- | --- |
|  | Right-sided origin | | Left-sided origin | | Right-sided origin | | Left-sided origin | |
|  | PVC | SR | PVC | SR | PVC | SR | PVC | SR |
| ALL | 74.2% | 25.8% | 82.6% | 17.4% | 80.2% | 19.8% | 88.5% | 11.5% |
| G1† | 58.9% | 41.1% | 79.3% | 20.7% | 78.3% | 21.7% | 94.5% | 5.5% |
| G2^‡^ | 79.6% | 20.4% | 85.2% | 14.8% | 80.7% | 19.3% | 87.3% | 12.7% |
| G3^§^ | 84.9% | 15.1% | 83.5% | 16.5% | 78.6% | 21.4% | 92.2% | 7.8% |
| G4^¶^ | 88.2% | 11.8% | 91.8% | 8.2% | N.A. | N.A. | N.A. | N.A. |

**Supplemental Table 3. Contribution rate of PVC and SR to the origin prediction for each cardiac rotation.**

^†^Patients with TZ < V3

^‡^Patients with V3 ≤ TZ ≤ V4

^§^Patients with V4 < TZ

^¶^Patients with bundle branch block

G, group; PVC, premature ventricular contraction; SR, sinus rhythm

**Supplemental Figure 1.**


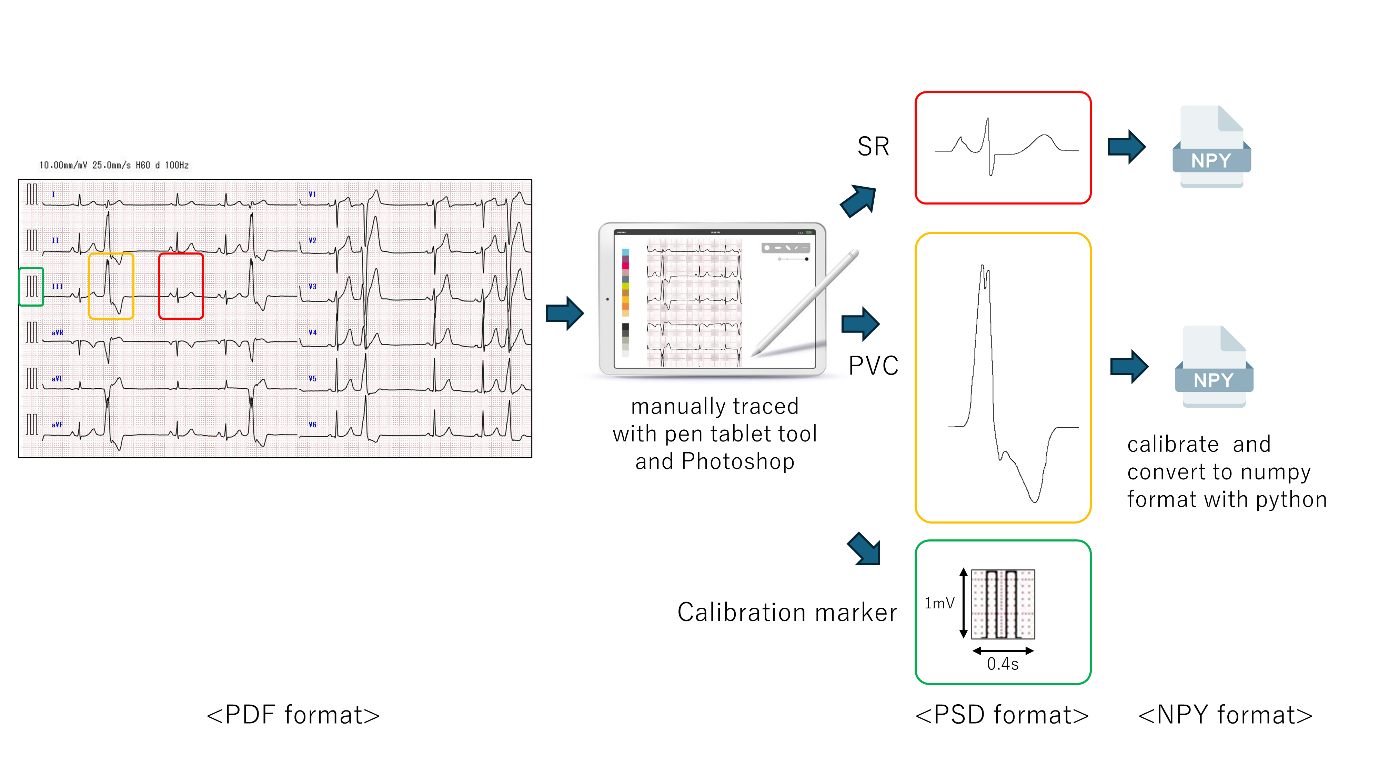


**Supplemental Figure 1. The manual tracing process to convert ECGs from PDF files into a format usable for deep learning models**. Out of the entire dataset, 303 cases were derived from PDFs, while 290 cases were acquired digitally. The cases derived from the PDF were manually traced using a pen tablet tool and Adobe Photoshop (version 24.1, Adobe Inc., San Jose, CA, USA). PVC, premature ventricular contraction; SR, sinus rhythm

**Supplemental Figure 2.**


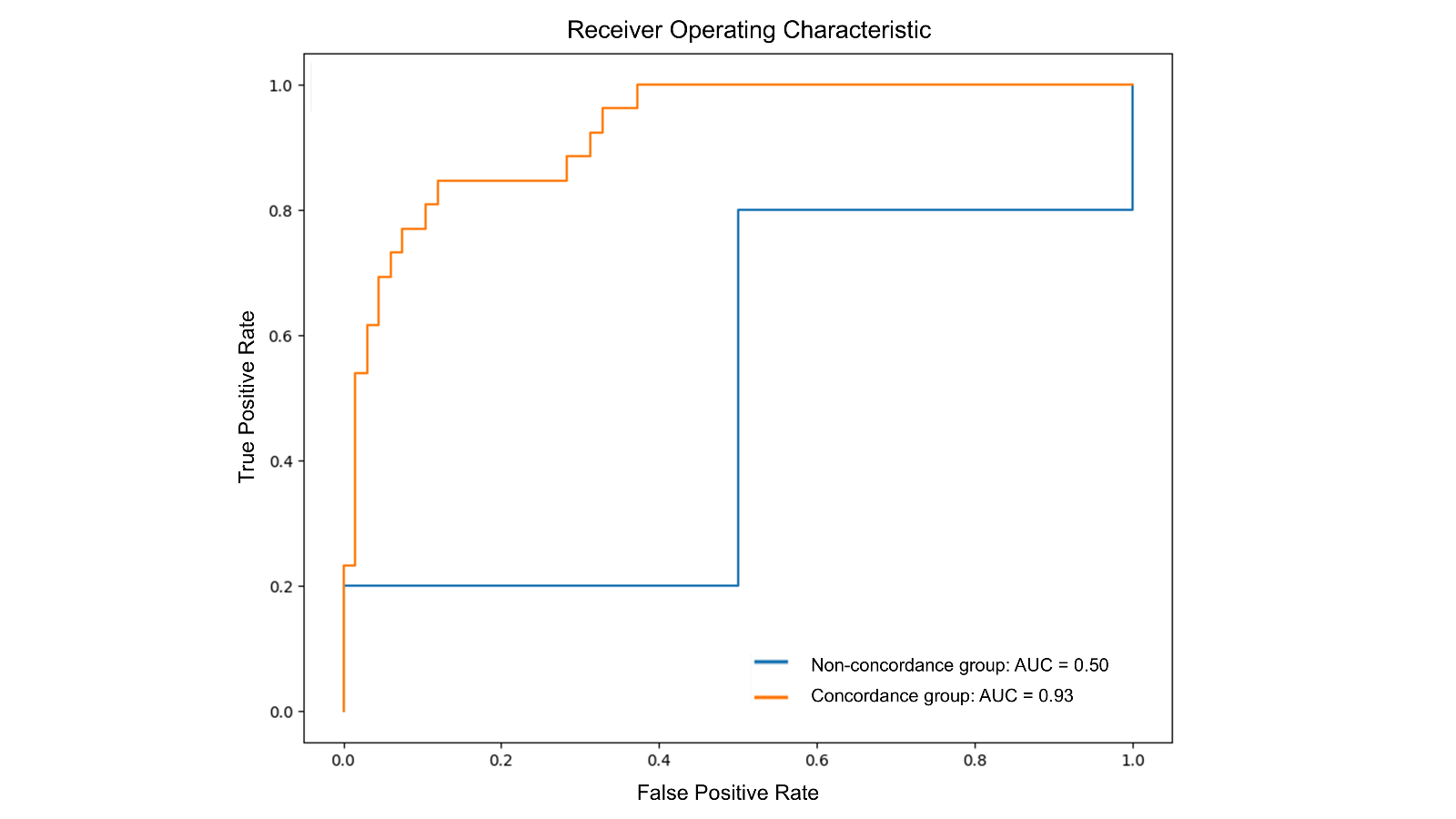


**Supplemental Figure 2. The receiver operating characteristic AUC for the Concordance and Non-concordance groups between the prematurity of QRS onset and response to ablation.** The AUC value for the concordance group was 0.93, while the AUC for the non-concordance group was 0.50. AUC, area under the curve.
